# Clinical cut-off scores for the Borderline Personality Features Scale for Children to differentiate among adolescents with Borderline Personality Disorder, other psychopathology, and no psychopathology: a replication study

**DOI:** 10.1186/s40479-024-00264-1

**Published:** 2024-08-26

**Authors:** Tess Gecha, Veronica McLaren, Carla Sharp

**Affiliations:** 1 University of Houston, Houston, TX USA; 2 Middlesex Health, Middletown, CT USA

**Keywords:** Research assessment, Borderline personality disorder, Sub-clinical borderline personality disorder, Adolescents, Borderline personality features scales for children

## Abstract

**Background:**

Despite being one of the most popular measures of borderline pathology in adolescents, only one study has evaluated clinical cut-off scores for the Borderline Personality Features Scale for Children (BPFS-C) using a small sample without a healthy comparison group (Chang B, Sharp C, Ha C. The Criterion Validity of the Borderline Personality Features Scale for Children in an Adolescent Inpatient Setting. J Personal Disord. 2011;25(4):492–503. 10.1521/pedi.2011.25.4.492.). The purpose of the current study was to replicate and improve on the limitations of the prior study conducted by Chang et al. to more definitively establish clinical cut-off scores for the self- and parent-report versions of the BPFS-C to detect clinical and sub-clinical borderline personality disorder (BPD) in a large sample of adolescents with BPD, other psychopathology, and no psychopathology.

**Methods:**

A total of 900 adolescents ranging from ages 12–17 participated in this study. The clinical sample consisted of 622 adolescents recruited from an inpatient psychiatric facility, and the healthy control sample consisted of 278 adolescents recruited from the community. All participants completed the BPFS-C and were administered the Child Interview for DSM-IV Borderline Personality Disorder (CI-BPD).

**Results:**

Using three-way ROC analyses, cut-off scores on the self- and parent-report versions of the BPFS-C distinguishing adolescents with BPD from those with subclinical BPD, and those with subclinical BPD from healthy adolescents were established.

**Conclusions:**

These findings support the use of both versions of the BPFS-C to detect adolescents with BPD and sub-clinical BPD.

## Background

Borderline personality disorder (BPD) is a serious psychiatric disorder marked by a pervasive pattern of affective instability, identity disturbance, impulsivity, and difficulties in interpersonal functioning [1]. Individuals with the disorder suffer from high levels of impairment and feelings of being a burden and are at high risk for suicide [2–4]. While previously thought to be mostly an adult disorder, research over the last two decades has shown that BPD is just as prevalent and valid in adolescents as in adults [5–9]. Community-based prevalence rates suggest that by 16 years of age, 1.4% of the population will meet criteria for BPD and by 22 years of age, 3.2% of the population will meet criteria for the disorder [10]. As clinical severity increases, so do the prevalence rates of BPD. Within clinical populations, roughly 11% of adolescent outpatients and between 19% and 53% adolescent inpatients meet for BPD [7, 11–13]. These numbers demonstrate that a substantial number of adolescents suffer from BPD, enough to warrant the development of reliable and valid assessment tools to aid in early diagnosis of such a serious psychiatric disorder. In response to this need, the Borderline Personality Features Scale for Children (BPFS-C) was created and has since become a popular measure of borderline pathology in adolescence [14].

The BPFS-C was created using age-adapted items from the Personality Assessment Inventory-Borderline Features Scale (PAI-BOR) [15], an already well-established measure for BPD in adults. The BPFS-C is a self-report measure that aims to assess four domains of borderline pathology, including affective instability, identity problems, negative relationships, and self-harm. Prior validation studies have shown that the measure has good to high internal consistency and good construct and criterion validity [1, 13, 14, 16]. The measure was also found to have high test-retest reliability and parent-child informant convergence and measurement invariance across sex and over time has been demonstrated [1, 14, 17, 18]. Studies examining its factor structure have shown mixed findings. Support has been demonstrated for a single factor, bi-factor, and four factor model [7, 16, 19]. On balance, given high correlations between factors, the general consensus that the total score on the BPFS-C rather than its subscale scores should be used [20].

To obtain the most comprehensive picture of an adolescent’s functioning, a parent-report version of the BPFS-C was adapted in 2011 (BPFS-P) [13]. Validation studies suggest that the parent-report has adequate internal validity as well as good criterion validity and measurement invariance across child age and gender and gender of caregiver informant [1, 13, 21]. The correlation between self- and parent-report versions of the BPFS has demonstrated modest to high agreement [1, 13].

Given the strong psychometric properties demonstrated for the BPFS, it has since been translated into French [16], Portuguese [16], and Chinese [17]. Further, the shortened 11-item version (BPFS-11) [19] has been translated into Italian [22] and Turkish [23]. Yet, only one study thus far has evaluated clinical cut-offs for the BPFS-C. Chang et al. [1] identified an optimal cut-off score of 66 for the BPFS-C and 72 for the BPFS-P using an adolescent inpatient sample. While informative, there are limitations to this study. First, the study sample size consisted of only 51 adolescents, including 20 inpatients with BPD and 31 psychiatric controls. Replication in a larger sample is necessary to confirm accuracy of clinical cut-offs. Second, the absence of a healthy control group is limiting because it compromises researchers’ ability to establish a normative baseline against which they can assess deviations. Including a healthy control sample also has the benefit of determining a clinical cut-off that indexes very low levels of borderline features (healthy young people), sub-threshold levels of borderline features (psychiatric controls without BPD) and those above clinical threshold who meet full criteria for BPD. Establishing a clinical cut-off for sub-threshold but at-risk young people opens avenues for evaluating unique correlates and trajectories associated with sub-threshold BPD in addition to correlates and trajectories of full-blown BPD. It also allows description of within-person variation around clinical threshold over time.

While the field of personality psychology is increasingly moving toward using dimensional approaches to conceptualize personality disorder (PD), there is still a wealth of knowledge available on BPD as it currently stands in Section II the Diagnostic and Statistical Manual of Mental Disorders (DSM-V) [2]. The number of studies assessing Criterion A, or level of personality functioning (LPF), of the Alternative Model for Personality Disorders (AMPD) in adolescence has increased significantly over the last several years [24–29]. However, the bulk of existing empirical research still focuses on BPD, and mental health services continue to be organized around the categorical diagnosis. As such, BPD remains relevant to study. Further, within the context of recent developments in the conceptualization of PD, BPD criteria has been found to represent the core features of personality disorder [30–32]. As such, it can be used as a useful proxy for LPF. With the dimensionalization of personality disorder, it is now also possible to conceive of personality disorder from a lifespan perspective [33], which places a new focus on early identification of personality disorder in young people [6]. Early identification enables clinicians to treat emerging personality pathology in adolescents before symptoms become fully syndromal. According to stepped care, which is possible through early detection, these individuals can be treated with less intrusive and less resource-intensive interventions that are necessary for more severe cases of psychopathology, lessening the burden on both patients and the mental healthcare system [6].

Against this background, there is still need for valid and reliable measures to assess for borderline pathology in adolescents. The BPFS is a particularly strong measure as it has probably the biggest literature base of all self-report measures of BPD in adolescents at present, demonstrated strong psychometric findings in both self- and parent-report versions, is relatively short in length (24 items) and easy to score (total score is the sum of all items, which require no reverse or mid-point scoring), and has been translated and validated in multiple languages [16–18]. Further, in its development, Crick et al. [14] rigorously considered developmental factors in developing and adapting items of the BPFS. However, the original paper establishing its cut-offs was based on a very small sample (*n* = 51). Moreover, clinical cut-offs were established by comparison with psychiatric controls only, raising the question as to what cut-offs are appropriate to distinguish healthy adolescents from those with personality disorder. As such, the present study aimed to replicate the Chang et al. [1] study establishing cut-off scores, correcting for its limitations and extending prior findings by using a larger sample and comparisons between healthy controls, psychiatric controls, and adolescents with BPD to establish clinical cut-off scores that discriminate the three groups from each other.

## Methods

### Participants

900 youth participated in the study, including a clinical sample (*n* = 622) and a sample of healthy controls (*n* = 278) recruited from the community. The Institutional Review Board (IRB) at University of Houston and the Institutional Review Board for Human Subject Research for Baylor College of Medicine and Affiliated Hospitals (BCM IRB) reviewed and approved study procedures.

The clinical sample consisted of 804 consecutive admissions to the adolescent unit of an inpatient psychiatric facility in a large metropolitan area in the southwestern United States. Consecutive admissions were screened for the following inclusion criteria: (1) being 12 to 17 years of age, and (2) sufficient fluency in English to complete all research. Exclusion criteria for study participation comprised the following: (1) diagnosis of schizophrenia or any psychotic disorder, and/or (2) diagnosis of mental retardation. One hundred and fifty-two adolescents were excluded based on inclusion/exclusion criteria, declining or revoking consent, or being discharged prior to assessment. Thirty adolescents did not have complete data for study measures, resulting in a final sample of 622 adolescents. Adolescents who did not complete all study measures were slightly younger than those who did (*t*(650) = 2.857, *p* = .004), but did not differ from those who did by gender (*χ*^2^(1) = 2.04, *p* = .154) or race (*χ*^2^(4) = 3.01, *p* = .556). BPD status among the remaining 622 adolescents was determined using the Childhood Interview for DSM-IV Borderline Personality Disorder (CI-BPD) [34], a semi-structured interview assessing DSM-IV criteria of BPD. 33% (*n* = 205) of the sample met full threshold criteria for DSM-IV BPD and constituted the BPD group, and the remaining 67% (*n* = 417) did not meet BPD criteria and constituted the psychiatric control (PC) group. We note that BPD criteria have not changed with the publication of the DSM-5 (2013). Other psychopathology of participants was determined using the Diagnostic Interview Schedule for Children (C-DISC) [35] and is described in Table 1.

**Table 1:** Demographic characteristics of each sample

	BPD (*n* = 205)		Psychiatric Controls (*n* = 417)		Community adolescents (*n* = 278)	
	***n *** **or M**	**SD or %**	***n *** **or M**	**SD or %**	***n *** **or M**	**SD or %**
Age	15.25	1.49	15.39	1.39	14.85	1.53
Gender						
Male	39	19.1	185	44.6	95	34.2
Female	165	80.9	230	55.2	183	65.8
Race						
Asian	5	2.7	15	4.1	52	2.0
African American	4	2.2	7	1.9	84	30.4
White	154	84.6	326	89.3	2	0.7
Hispanic	3	1.6	3	0.8	74	26.8
Multiracial/other	16	8.8	15	4.1	21	7.6
Psychopathology						
YSR T-score	71.47	8.15	62.48	9.65	50.30	10.22
CBCL T-score	70.22	6.20	67.91	6.40	42.33	7.89
Diagnosis						
Depressive	134	73.2	190	50.0		
Bipolar	26	14.2	15	3.9		
Eating disorder	28	15.2	22	5.8		
Externalizing	110	59.8	131	34.4		
Anxiety	135	73.0	199	52.0		
Substance Abuse	21	36.2	40	30.3		
CI-BPD Total	14.01	2.07	5.67	3.27	0.87	1.76

Percentages are calculated out of those reporting for each characteristic.

The community, or healthy control (HC), sample consisted of 323 adolescents recruited from public schools and community organizations in a large metropolitan area in the southwestern United States. Inclusion criteria for study participation consisted of: 1) being 12–17 years of age and 2) sufficient fluency in English to complete all research. Exclusion criteria consisted of: 1) meeting criteria for any psychological disorder, which was determined through self-report of current or prior diagnosis with a psychiatric disorder. Forty-five adolescents did not have complete data for study measures, resulting in a final sample of 278 adolescents. Adolescents who did not complete all study measures were slightly older (*t*(321)=-4.54, *p* < .01), more likely to be African American or White, and less likely to be Hispanic (χ^2^(4) = 43.18, *p* < .01) than those who did, but did not differ from those who did by gender (χ^2^(1) = 0.247, *p* = .619). Gender, age, and ethnicity information for all groups is presented in Table 1.

### Borderline personality features scale for children - child and parent report

The Borderline Personality Features Scale for Children (BPFS-C) [14] is a 24-item self-report instrument that assesses borderline personality features among children and adolescents aged nine and older. The BPFS-C is based upon the PAI-BOR and modified for youth. A four-point Likert scale ranging from 1 (*not at all true*) to 5 (*always true*) is used to report on affective instability, identity problems, negative relationships, and self-harm with higher scores indicating higher levels of borderline features. The measure has demonstrated good validity, internal consistency, and test-retest reliability [14]. In 2011, Sharp et al. developed the parent-report version using the same items adapted for parent report (BPFS-P) [13]. The BPFS-P has demonstrated adequate internal consistency for both total and subscale scores [1].

#### Borderline diagnosis

BPD status against which clinical cut-offs could be determined was evaluated in the clinical adolescents using the Childhood Interview for Borderline Personality Disorder (CI-BPD) [34]. The CI-BPD is an interview-based measure that probes each of the nine diagnostic criteria for BPD in section II of the DSM-5. Each criterion is rated on a 0–2 scale (0 if symptom is absent, 1 if symptom is probably present, and 2 if the symptom is definitely present). A score of 2 on five or more criteria (total score of at least 10) indicates a BPD diagnosis while a sub-threshold score of 2 on three or four criteria (total score of 6 or 8) has been used in previous studies [36, 37]. Item scores were summed to compute a total score, which ranged from 0 to 18. The CI-BPD has shown good inter-rater reliability and internal consistency, and associations with other measures of BPD symptomatology, suggesting strong external validity [12]. In this study, CI-BPD served as the grouping variable, with those meeting at least five of nine criteria falling into the BPD group. In the current sample, Cronbach’s alpha for the CI-BPD was 0.86. Of the inpatient sample, 107 of 622 participants (17.2%) were coded by a second (blinded) coder for reliability purposes, and inter-rater reliability was found to be acceptable (Kappa = 0.74).

### Statistical analyses

Analyses were carried out in SPSS for Windows, version 26 [38] and the R 3.5.1 software [39]. First, demographic characteristics were evaluated for each diagnostic group. Differences between groups on categorical demographic data were evaluated using χ^2^ tests, and differences on continuous measures were evaluated using one-way analysis of variance (ANOVA). Chi-square tests were also performed to examine the relationship between dummy variables distinguishing adolescents identified through self-report vs. parents as having BPD (0 = below identified cut point, 1 = above identified cut point). Tukey’s test was used for post-hoc comparisons. The correlation between the BPFS-C and BPFS-P was calculated using Pearson correlation analysis. Reliability was calculated using Cronbach’s alpha. To test for age effects, independent samples t-tests were conducted using a dummy variable (0 = early adolescents, 1 = late adolescents) that was created to distinguish early adolescents (ages 12–14) from late adolescents (ages 15–17).

Receiver operating characteristic (ROC) analyses were used to determine screening ability and optimal cutoff points of the BPFS-C and BPFS-P for both dichotomous and multi-group classification. For dichotomous screening, a ROC curve is obtained by plotting the sensitivity of a test against its specificity on a graph. The area under the curve (AUC) is thus a measure of diagnostic accuracy of the test; at an AUC of 0.5, classification accuracy is at chance-levels, while an AUC of 1.0 is perfect classification [40]. An AUC < 0.7 suggests low diagnostic accuracy, from 0.7 to 0.9 moderate accuracy, and > 0.9 high accuracy [41]. Screening ability was computed to distinguish between BPD versus non-BPD (PC and HC) and between BPD versus PC. Optimal cut points were determined by the intersect point of sensitivity and specificity, following the methods used by Chang et al. [1].

Multi-category classification extends these ideas to the instances with more than two diagnostic categories by using a ROC surface, in which a three-dimensional ROC surface is obtained by plotting the sensitivity, specificity, and true discovery rate [42, 43]. The volume under the surface (VUS) can be used as an indicator of the diagnostic accuracy of the test for discrimination between more than two groups. Like the AUC, a VUS of 1.0 indicates perfect classification accuracy. A VUS of 0.536 or above is considered satisfactory [44]. Optimal cut-off points can be determined by finding the coordinate on the ROC surface that minimizes the distance to the perfect classification point.

## Results

### Demographic information

Demographic information is summarized by group and is described in Table 1. The healthy control group was significantly younger than both clinical groups (*F*(2,893) = 11.52, *p* < .01). There was a significantly higher proportion of males in the PC group versus the HC group and in the HC group versus the BPD group (*χ*^2^(2) = 39.02, *p* < .01). Additionally, the racial composition of the HC group differed significantly from the clinical groups (*χ*^2^(8) = 456.17, *p* < .01); there was a higher proportion of African American, Asian, and Hispanic participants and a lower proportion of White participants in the healthy controls group than in either of the clinical groups. Both self-report (*F*(2, 855) = 214.61, *p* < .01) and parent-report (*F*(2,828) = 417.96, *p* < .01) BPFS scores were higher for the BPD group than the PC group, and higher for the PC group than the HC group. Likewise, CI-BPD scores were higher for the BPD group than the PC group, and higher for the PC group than the HC group (*F*(3, 900) = 1010.52, *p* < .001).

[Table 1]

### Informant convergence and consistency

The BPFS-P and BPFS-C were significantly positively related (*r* = .52, *p* < .01). Internal consistency was good for both the BPFS-C (α = 0.91) and the BPFS-P (α = 0.95).

### Age effects

Results from independent t-tests did not yield any significant differences between early and late adolescents on BPFS-C, BPFS-P, or CI-BPD mean total scores (BPFS-C: *t*(855) = 0.269, *p* = .788; BPFS-P: *t*(829) = − 0.232, *p* = .817; CI-BPD: *t*(897) = 0.328, *p* = .328).

### Discriminating dichotomous groups

**Fig. 1:**
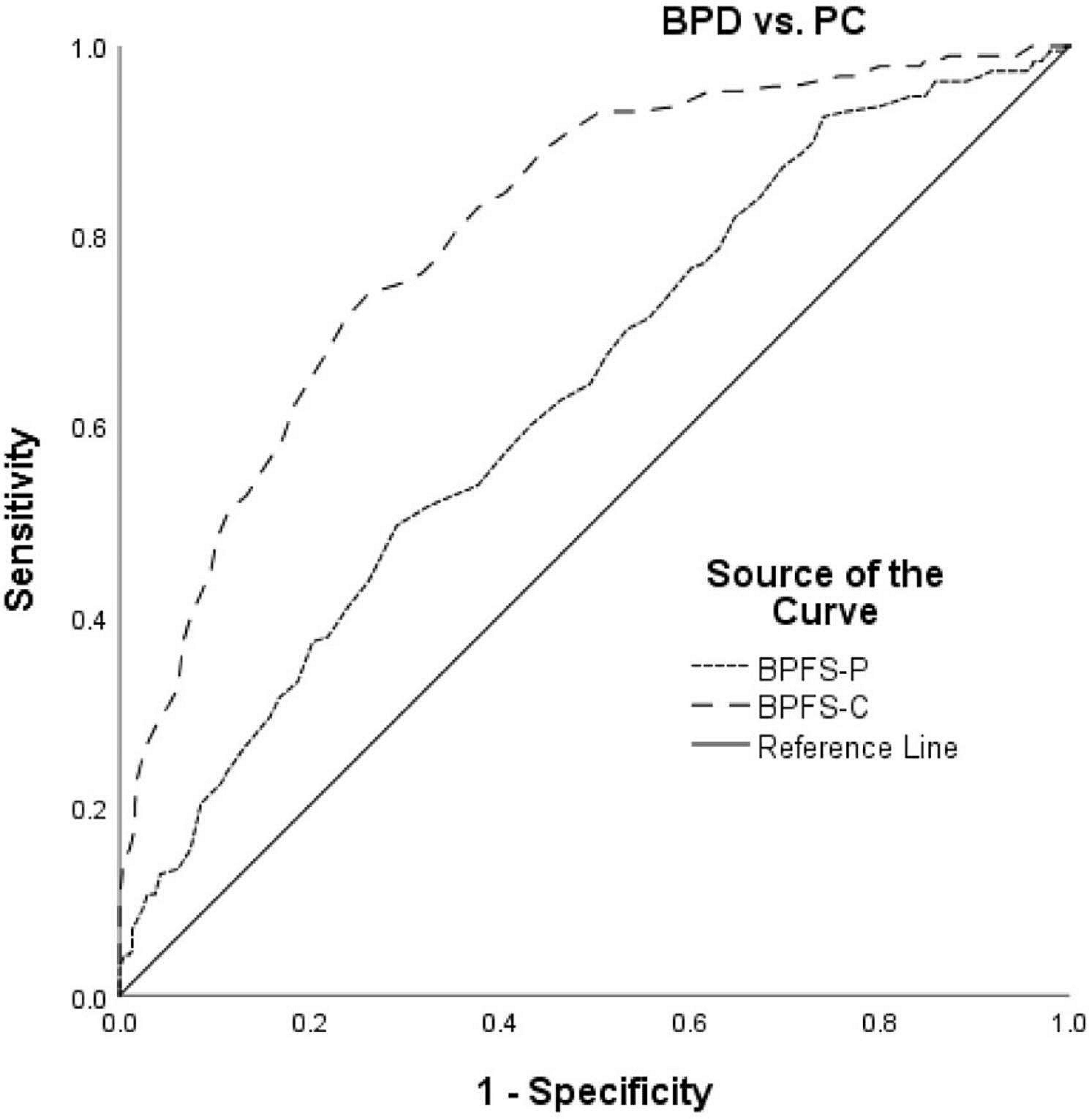
Receiver operating characteristic (ROC) curve for the dichotomous screening of BPD versus PC for the BPFS-C and BPFS-P

**Fig. 2:**
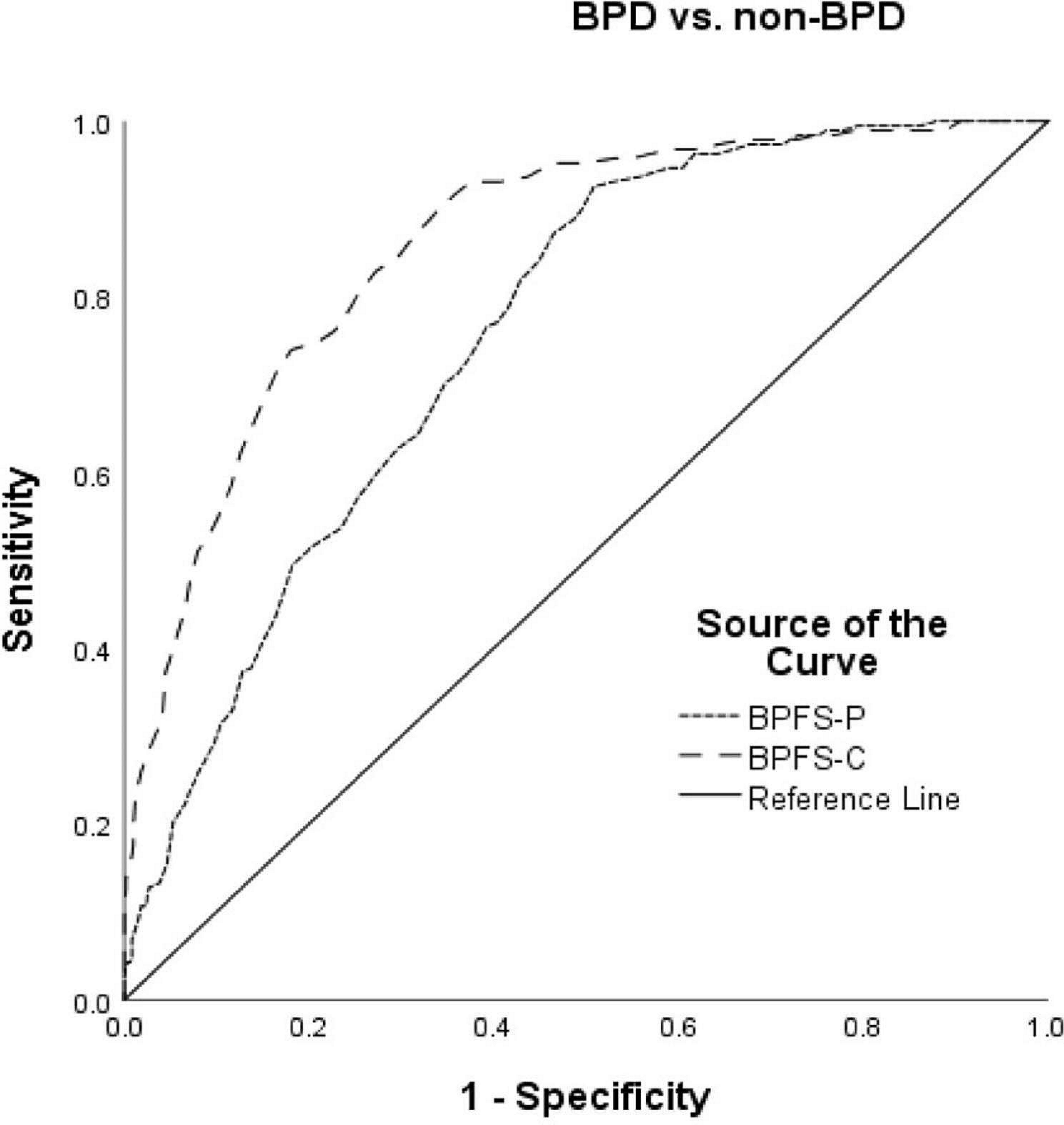
Receiver operating characteristic (ROC) curve for dichotomous screening of BPD versus non-BPD for BPFS-C and BPFS-P

ROC curves were drawn for BPD versus PC replicating the analyses used by Chang et al. [1], as well as for BPD versus non-BPD, to determine the ability of each form of the BPFS to discriminate between the two groups (Figs. 1 and 2). For BPD versus PC, the BPFS-C (AUC = 0.81) demonstrated moderate diagnostic accuracy, with the value lying between 0.70 and 0.90 which indicates moderate and excellent diagnostic accuracy, respectively. The BPFS-P (AUC = 0.64) demonstrated low diagnostic accuracy, although we note that the value is not far below the 0.70 suggested cut-off for moderate diagnostic accuracy. For the BPFS-C, the optimal cut point was 75, which yielded a sensitivity of 0.74 and a specificity of 0.74, indicating that at least 74% of young people would be correctly identified with the measure. This is higher than the cut point of 66 identified by Chang et al. [1]. For the BPFS-P, the optimal cut point was 74, which yielded a sensitivity of 0.60 and a specificity of 0.57, indicating that 60% of young people would be correctly identified with this measure. This cut point is similar to the one identified by Chang et al. [1].

For BPD versus non-BPD, both the BPFS-C (AUC = 0.86) and the BPFS-P (AUC = 0.76) had moderate diagnostic accuracy, with the BPFS-C approaching excellent diagnostic accuracy. The optimal cut point for the BPFS-C was 72, which yielded a sensitivity of 0.78 and a specificity of 0.76, meaning that 78% of young people would be correctly identified. Values were again lower for the BPFS-P, with an optimal cut point of 71 (sensitivity 0.68; specificity 0.67). A chi-square test was used to evaluate the extent to which the same individuals were identified with the child and parent version. A chi-square test using dummy variables representing those above and below the 72 cut point for self-report and those above and below the 71 cut point for parent report produced a significant test statistic, *X*^2^ (1, *N* = 900) = 98.0, *p* < .001, with a 56.4% overlap between adolescent and parent report.

### Discriminating three groups

**Fig. 3:**
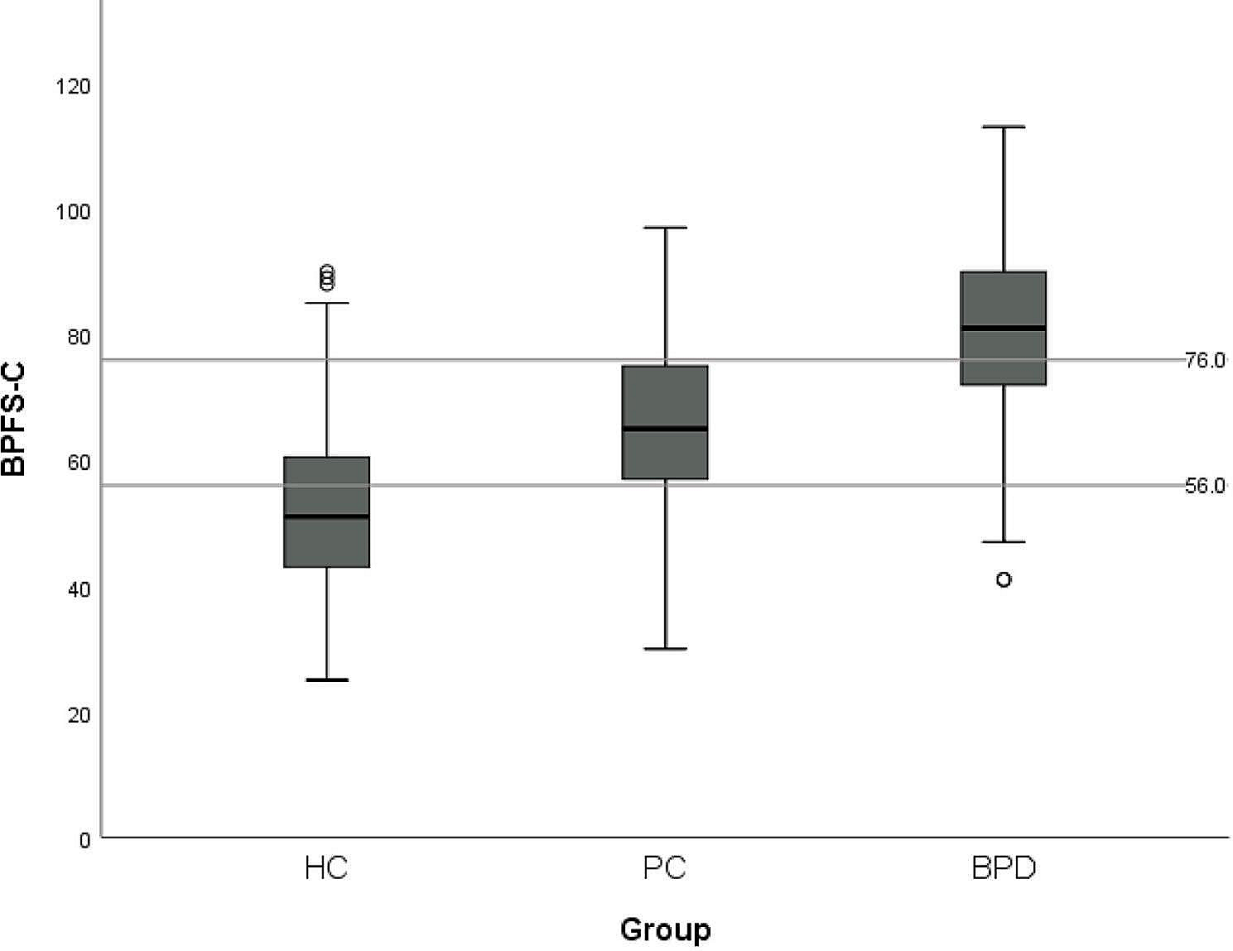
Optimal cut points of the BPFS-C for HC, PC, and BPD

**Fig. 4:**
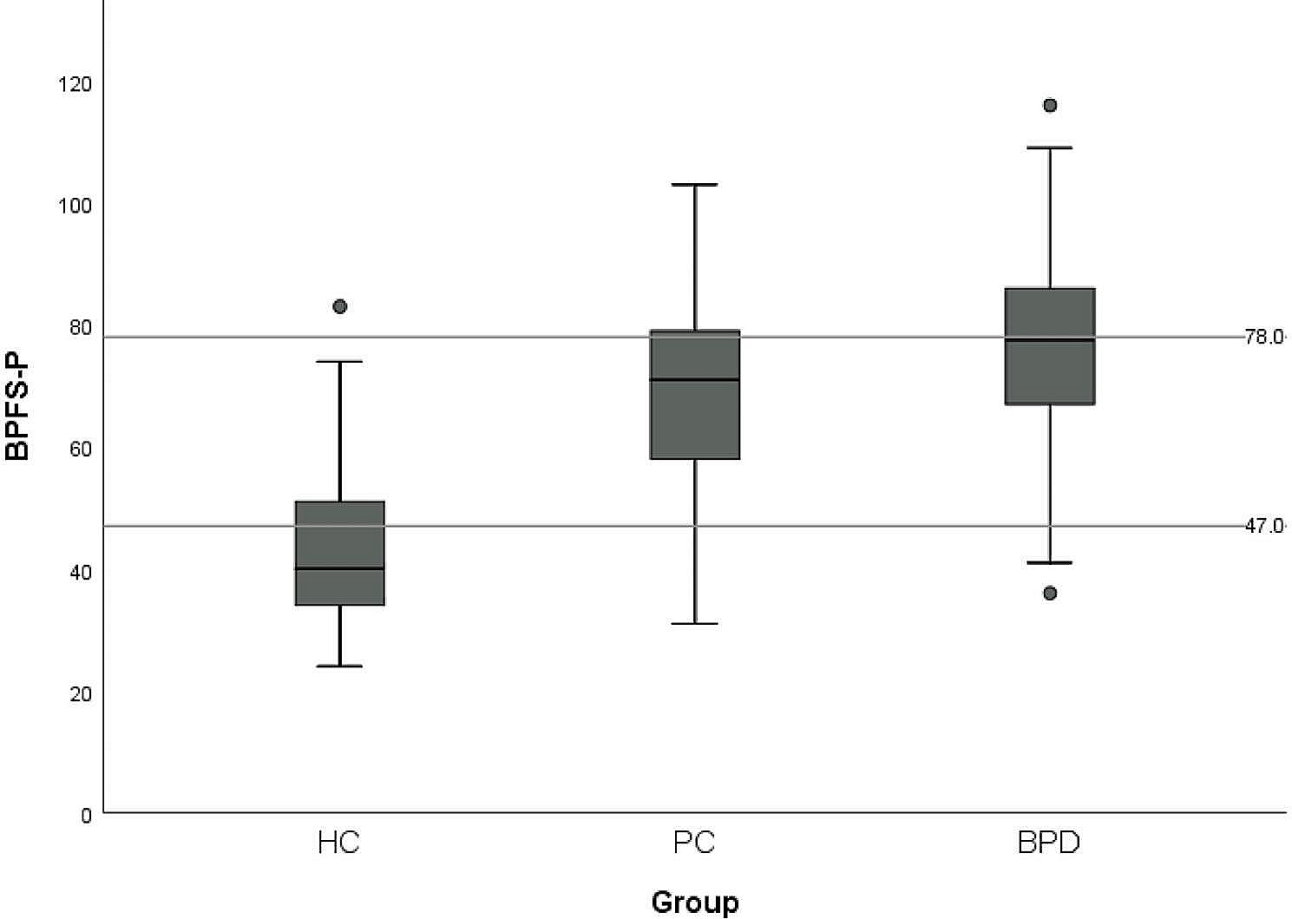
Optimal cut points of the BPFS-P for HC, PC, and BPD

The ROC surfaces for both the BPFS-C and the BPFS-P as a measure for discriminating HC, PC, and BPD groups were analyzed. The VUS for the BPFS-C was estimated as 0.55 (95% CI, 0.51-0.59), indicating satisfactory classification accuracy. The derived optimal cut points were 56 and 76, resulting in 60.4% of cases being correctly identified (Fig. 3). The VUS for the BPFS-P was estimated at 0.57 (95% CI, 0.52-0.61), indicating satisfactory classification accuracy. The derived optimal cut points were 47 and 78, resulting in 63.6% of cases being correctly identified (Fig. 4). The chi-square test using dummy variables representing those above and below the cut points for self-report and parent report produced a significant test statistic, *X*^2^ (1, *N* = 900) = 107.0, *p* < .001, with a 56.7% overlap between adolescent and parent report.

## Discussion

The current study aimed to expand upon findings from the Chang et al. [1] study by identifying clinical cut-off scores on both the BPFS-C and BPFS-P, correcting for limitations by using a larger sample that includes healthy and psychiatric controls and adolescents with BPD. Findings show that both self- and parent-report versions of the BPFS can discriminate adolescents at clinical threshold as well as below clinical threshold compared to healthy controls. In line with previous research, both measures demonstrated good internal consistency [45]. Further, the measures demonstrated good informant convergence with a moderate to large effect size (*r* = .52), which is similar to findings from the Chang et al. [1] study.

ROC analyses showed that a score of 75 on the BPFS-C distinguished psychiatric controls from adolescents with BPD, which is nine points higher than the cut-off score of 66 identified by Chang et al. [1]. On the BPFS-P, a score of 74 was found to discriminate psychiatric controls from adolescents with BPD, which is two points higher than the cut-off score of 72 identified by Chang et al. [1]. The self- and parent- report versions’ cut-off scores, 75 and 74 respectively, identified in our study are better aligned than the previous identified cut-off scores of 66 and 72, suggesting that the clinical cut-off established by Chang et al. was too low and may have led to false positives. The reason for the difference in identified cut-off scores between the two studies could be due to the considerably small sample size that was used by Chang et al. [1], which could have led to inaccuracies in the estimated validation parameters associated with ROC analysis [45]. With respect to the study’s novel BPD versus non-clinical comparison, both self and parent versions of the measure demonstrated moderate accuracy and yielded cut-off scores of 72 and 71 respectively. Since age-stratified differences were not identified, there was no need to conduct separate ROC analyses for early and late adolescents, and identified cut-off scores can be applied to both age groups. The 56.4% overlap between adolescent and parent report suggests that discrepancies exist between parent-and self-report, further suggested by the moderate correlation (> 0.50) between parent and self-report. Discrepancies in parent and youth self-report are common and correlation coefficients often do not exceed 0.30, so the moderate association found here can be considered comparatively strong. Discrepancies have been viewed as a strong clinical indicator in psychopathology more generally and personality pathology specifically [46, 47].

A novel aspect of our study was the three-group comparison, which established clinical cut-off scores distinguishing adolescents who may be at risk of developing BPD and those meeting full BPD criteria from a healthy population. Results show that both self- and parent-report versions of the measure demonstrated satisfactory classification accuracy when distinguishing the three groups. The self-report version was found to have optimal cut-off scores of 56 and 76 and the parent-report version was found to have optimal cut-off scores of 47 and 78, with moderate informant agreement.

The results of this study can be interpreted against the backdrop of several recent trends in the field of personality disorder. Despite abundance of research legitimizing the emergence of borderline pathology in adolescents, clinicians continue to be reluctant to diagnose BPD in young people [9, 48]. Many, but not all, clinicians continue to believe that those with BPD are less worthy of care, and that it is pointless to try to treat them as they are hopeless cases [49]. In reality, high levels of symptomatic remission have been found in borderline populations [50, 51], a finding that should encourage clinicians to be less resistant to making the diagnosis and engaging in treatment to help these individuals who capable of achieving remission and recovery during their lifetime.

Cut-off scores and measures like the BPFSC will help clinicians identify the most appropriate level of care for adolescents. The appropriate treatment for an adolescent with subthreshold BPD looks very different from the treatment of an adolescent with BPD. Discriminating these adolescents using the measure’s cut-off scores identified in this study will save the families of adolescents with sub-threshold BPD time and resources that are required of more intensive treatment. Considering the legitimacy of the disorder in adolescents and its treatable nature, clinicians are in need of valid tools that can assist in diagnosis that will help inform treatment. This study identifies the BPFSC as such a measure, which is easy to administer and time-efficient in clinical settings.

Adolescence represents an opportune time for diagnosis so that adolescents can receive the appropriate treatment to impede the progression of borderline pathology and impairment associated with it [50]. Further, it is essential to discriminate adolescents with lower, but still clinically relevant levels of BPD symptoms. Accordingly, adolescents with subthreshold BPD have been shown to fare worse socially and occupationally and are more likely to be referred for suicidal ideation/behavior than clinical comparisons without any BPD features [52], and suffer from a significant number of psychiatric symptoms and social difficulties that are roughly equivalent to those experienced by adolescents meeting criteria for one personality disorder [53], and are rated as equally depressed by clinicians when compared to those meeting full criteria for BPD [54]. These findings show that there is a significant need for measures that accurately detect individuals with subsyndromal BPD as they too suffer from high degrees of distress and impairment, and our findings provide strong support for the use of the BPFS-C and -P for this purpose.

While there is still value in assessing BPD, we recognize several benefits to be gained by shifting toward a more dimensional model of personality disorder (PD) [55] specifically the Alternative Model for Personality Disorder (AMPD). Understanding PD in terms of self and interpersonal functioning reduces stigma, provides a malleable treatment target, eliminates the issue of high comorbidity among PDs, and reduces risk of not detecting subthreshold individuals who need treatment. While there is clear value in assessing measures that adhere to this more nuanced conceptualization of PD, measures that assess borderline pathology in adolescents are still useful as there is still hesitancy among clinicians to discard of the BPD diagnosis in favor of new dimensional conceptualizations. As clinicians continue to work toward the dimensionalization of PD, BPD remains a specifier (for e.g. in the ICD-11), and the need for valid and reliable measures to assess the construct remains.

Limitations of this study includes that the racial demographics of the samples were discrepant, specifically between BPD and psychiatric control groups and community adolescents. Specifically, the BPD and psychiatric control samples were predominantly White, which was not mirrored in the community adolescent sample. Thus, the generalizability of our findings to other racial groups is limited. Further, comparing groups with such different racial makeups may have affected our observed results. As such, we recommend that future research be conducted on the cross-racial generalizability of the measure. Another limitation is the difference in sample sizes. The psychiatric control sample was much larger than the BPD and community adolescent samples. The disparity in sample sizes might reduce reliability of our results. Participants from the clinical samples were mostly White and from families of high socioeconomic status with college-educated, married parents who were able to access services from private psychiatric hospitals. In this way, findings may not generalize to adolescents from families with different demographic characteristics. Finally, generalizability to other, less severe clinical samples is limited as our clinical sample consisted of adolescents from an inpatient unit.

## Conclusions

Taken together, our findings suggest that the BPFS-C and BPFS-P are valid and reliable measures that can be used to discriminate adolescents with BPD. Being able to accurately identify these individuals is essential to ensuring that they receive the most appropriate care that will put them in the best position for recovery. Our findings further demonstrate how the measure can also be useful in identifying adolescents with subclinical levels of borderline pathology, paving the way for clinicians to identify and treat these individuals early on before their symptoms reach clinical threshold.

## Data Availability

No datasets were generated or analysed during the current study.

## Abbreviations

BPD: Borderline personality disorder
PC: Psychiatric control
HC: Healthy control
ROC: Receiver operating characteristic
AUC: Area under the curve
VUS: Volume under the surface
